# A case of concurrent follicular lymphoma and lung cancer requiring differentiation from lymph node metastasis

**DOI:** 10.1111/1759-7714.15279

**Published:** 2024-03-13

**Authors:** Yutaka Takahara, Sumito Nagae, Aika Yamagata, Yoshihito Iijima, Akihiro Shioya, Sohsuke Yamada, Hidetaka Uramoto

**Affiliations:** ^1^ Department of Respiratory Medicine Kanazawa Medical University Kahoku‐gun Japan; ^2^ Department of Thoracic Surgery Kanazawa Medical University Kahoku‐gun Japan; ^3^ Department of Pathology and Laboratory Medicine Kanazawa Medical University Kahoku‐gun Japan

**Keywords:** follicular lymphoma, lung cancer, lymph node metastasis, multiple cancers

## Abstract

Lung cancer complicated by follicular lymphoma has rarely been reported in the literature. A 69‐year‐old male with an abnormal shadow on a chest radiograph was referred to our hospital. A mass in the right lung was seen on chest computed tomography (CT). Positron emission tomography‐CT showed fluorodeoxyglucose accumulation in the esophagus and multiple intra‐abdominal lymph nodes, in addition to the right lung lesion. The lung lesion was diagnosed as a pulmonary adenocarcinoma after biopsy. Upper and lower gastrointestinal endoscopies did not reveal the presence of a tumor. Open lymph node biopsy was performed to determine the course of treatment, leading to a diagnosis of follicular lymphoma. The patient finally underwent radical resection for lung cancer; the follicular lymphoma was judged to be low‐grade and was followed up. When complications involving other organs are detected during systemic examination of a patient with lung cancer, it is necessary to distinguish between metastasis to other organs and complications of other malignant diseases, as this will greatly influence the treatment strategy.

## Graphical abstract

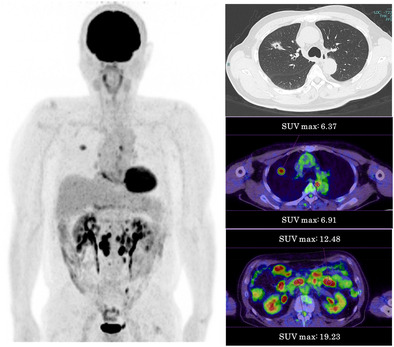

## INTRODUCTION

When malignant tumors overlap, it is necessary to evaluate each overlapping disease when treating patients. Lung and follicular lymphomas rarely coexist. Lung cancer is, however, the most common second primary malignancy in patients with lymphoma.^1^, ^2^, ^3^ We encountered a patient with lung cancer and multiple enlarged intra‐abdominal lymph nodes. Although distant lymph node metastasis of lung cancer was suspected, follicular lymphoma was diagnosed based on an open lymph node biopsy. Consequently, radical surgery was performed for the lung cancer. Clinicians should be aware that in patients with lung cancer with features suggestive of intra‐abdominal lymph node metastasis, there may be cases of combined lung cancer and lymphoma. We report this case to emphasize the need for accurate diagnosis to determine appropriate treatment strategies for such cases.

## CASE REPORT

A 69‐year‐old male was referred to our hospital for a simple chest radiograph, which revealed an abnormal shadow. There were no abnormal physical findings in the cardiopulmonary system or palpable lymph nodes on the body surface.

**FIGURE 1:**
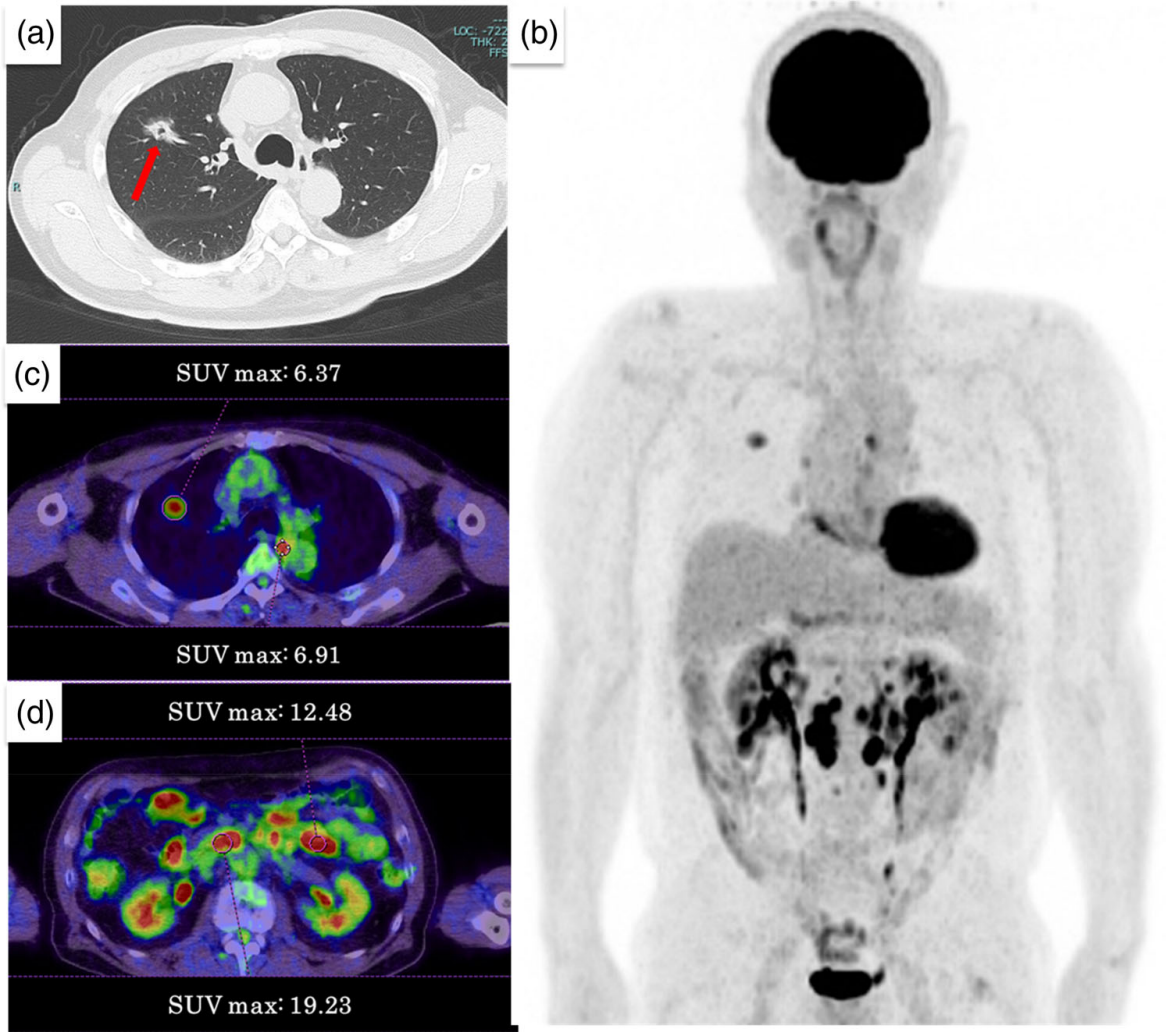
Computed tomography (CT) and fluorodeoxyglucose (FDG)‐positron emission tomography (PET)‐ CT image of the chest. (a) A plain CT scan of the chest showed a 27‐mm‐sized, part‐solitary, frosted shadow in the S3 of the right upper lobe (arrow). There was no evidence of enlarged hilar or mediastinal lymph nodes or pleural effusion. FDG‐PET showed FDG accumulation in the right lung mass, esophagus, and several intra‐abdominal lymph nodes (b). Maximum standardized uptake value (SUV) max was 6.37 for lung lesions (c), 6.91 for the esophagus (c), and 19.23 for the abdominal lymph nodes (d).

A computed tomography (CT) scan of the chest (Figure 1a) showed a 27 mm‐sized, part‐solitary, frosted shadow in S3 of the right upper lobe. Fluorodeoxyglucose‐positron emission tomography (FDG‐PET) (Figure 1b,c) showed FDG accumulation in the right lung mass, esophagus, and several intra‐abdominal lymph nodes.

Pretreatment diagnosis suspected lung cancer, esophageal cancer, or abdominal lymph node metastasis of esophageal cancer. Bronchoscopic lung biopsy was performed on the S3 tumor in the right lung upper lobe for a definitive diagnosis. The patient was diagnosed with adenocarcinoma based on the pathohistological examination.

Upper and lower gastrointestinal endoscopies revealed no gastrointestinal tumors. Capsule endoscopy revealed no mucosal lesions in the small intestine, although there were scattered extramural compressions that may have been caused by the enlarged lymph nodes.

**FIGURE 2:**
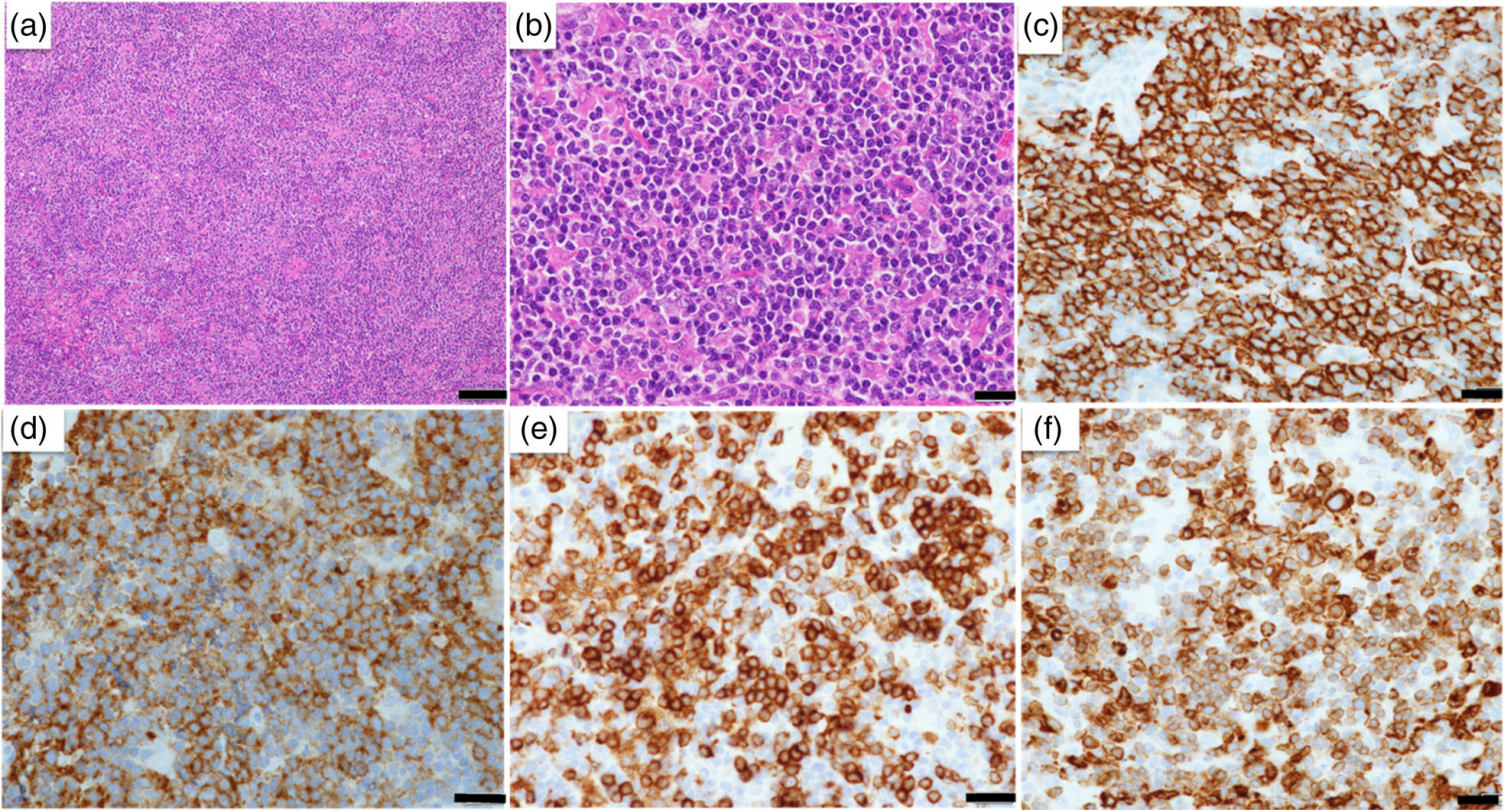
Histological examination of intra‐abdominal lymph nodes. The tumor had an ill‐defined lymph follicular architecture and proliferated medium‐sized atypical, lymphocyte‐like cells. Immunohistochemical findings were positive for CD20, CD10, CD79α, and Bcl‐2. (a, b) Hematoxylin and eosin (H&E) staining (a: scale bar = 100 μm; b: scale bar = 20 μm). (c) CD20 staining (scale bar = 20 μm). (d) CD10 staining (scale bar = 20 μm). (e) CD79α staining (scale bar = 20 μm). (f) Bcl‐2 staining (scale bar = 20 μm).

For a definitive diagnosis, the patient underwent an open intra‐abdominal lymph node biopsy. Histopathological findings (Figure 2) showed that the tumor had an indistinct lymph follicular architecture and proliferated medium‐sized atypical lymphocyte‐like cells. Immunohistochemical findings were positive for CD20(+), CD10(+), CD79α(+), Bcl‐2(+), and B cell markers; hence, follicular lymphoma was diagnosed.

**FIGURE 3:**
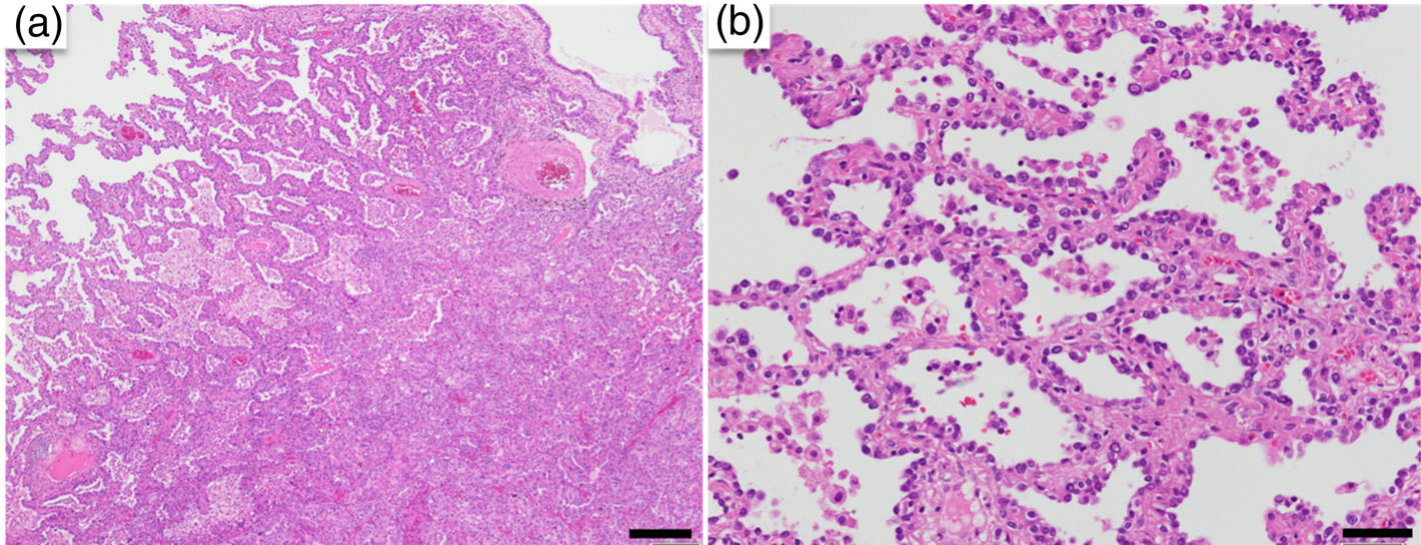
Histological examination of the lung tumor (H&E staining). Tumor cells proliferating in a papillary fashion were observed (a: scale bar = 200 μm; b: scale bar = 50 μm).

We concluded that the patient had stage II follicular lymphoma (Ann Arbor classification 2) and stage I primary lung cancer. After consultation with the hematology/oncology department, it was determined that the follicular lymphoma was stable and conservative follow‐up was feasible. Therefore, the patient underwent right upper lobectomy and hilar mediastinal lymph node dissection for lung cancer treatment. Histopathological findings (Figure 3) showed papillary growth of tumor cells. There was no invasion of the vascular system or pleura, and no lymph node metastasis. The pathological diagnosis was stage IB.

To date, the follicular lymphoma has not worsened since the lung cancer surgery. However, the FDG‐accumulating lesion in the middle esophagus noted by FDG‐PET is scheduled for continued monitoring by the Departments of Hematology/Oncology as it may be a lymph node lesion of malignant lymphoma.

## DISCUSSION

Abdominal lymph node metastases are relatively common in patients with non‐small cell lung cancer (NSCLC).^4^ In this case, the abdominal lymph node lesion was indicated by FDG accumulation due to malignant lymphoma but could have been overdiagnosed as lymph node metastasis of lung cancer. The combination of lymphoma and lung cancer is rare, with only scattered case reports.^5^, ^6^, ^7^ Therefore, the association between lung cancer and lymphoma remains inconclusive.^8^ Lung cancer is, however, the most common second primary malignancy in patients with lymphoma.^1^, ^2^, ^3^

In principle, it is desirable to treat malignant tumors with higher grades of malignancy first, in case of overlapping cancers. Follicular lymphoma is a representative low‐grade malignant lymphoma that accounts for 35% of all non‐Hodgkin's lymphomas and is often diagnosed after symptoms have advanced.^9^ Even so, patients with advanced stage follicular lymphoma require immediate treatment only when they have symptomatic lymph node or extralymphatic involvement, B symptoms, or thrombocytopenia.^9^

With the introduction of rituximab, an anti‐CD 20 antibody, the median overall survival of patients with follicular lymphoma has approached 20 years.^10^ The 10‐year survival rate of patients with follicular lymphoma in their 60s is approximately 78%.^9^ In contrast, the 5‐year survival rates for operable cases of lung cancer are 91.6%, 81.4%, 74.8%, 71.5%, 60.2%, and 58.1% for IA1, IA2, IA3, IB, IIA, and IIB, respectively; the prognosis worsens with each stage of disease progression.^11^

This case was an overlap of low‐grade (grade 2) follicular lymphoma and lung adenocarcinoma; from a prognostic standpoint, we believed that treatment of the lung adenocarcinoma should have been prioritized. However, there are few reports on the concurrent development of NSCLC and malignant lymphoma and no standard guidelines for treating this condition. Considering the slow clinical course of most follicular lymphomas, we expect to encounter similar cases in the future. We believe that this report provides suggestions for the future treatment of similar cases.

In summary, patients with lung cancer and enlarged intra‐abdominal lymph nodes, inaccessible to biopsy, may face a difficult choice of treatment. In this case, an open lymph node biopsy enabled the diagnosis of a combination of follicular lymphoma and lung cancer, and concordant treatment. Even in lung cancer cases complicated by other malignant diseases, it is necessary to assess the status and course of the concurrent disease to optimize suitable treatment options.

## AUTHOR CONTRIBUTIONS

All authors had full access to the study data and take responsibility for the integrity of the data and the accuracy of the data analysis. All the authors have read and approved the submitted version of the manuscript. Conceptualization: Yutaka Takahara. Resources: Yutaka Takahara, Sumito Nagae, Aika Yamagata, Yoshihito Iijima, Akihiro Shioya, Sohsuke Yamada, and Hidetaka Uramoto. Investigation: Yutaka Takahara, Sumito Nagae, Aika Yamagata, Yoshihito Iijima, Akihiro Shioya, Sohsuke Yamada and Hidetaka Uramoto. Writing–original draft preparation: Yutaka Takahara with support from Sohsuke Yamada and Hidetaka Uramoto.

## CONFLICT OF INTEREST STATEMENT

The authors have no actual or potential conflicts of interest to declare.
